# Overexpression of *BIT33_RS14560* Enhances the Biofilm Formation and Virulence of *Acinetobacter baumannii*

**DOI:** 10.3389/fmicb.2022.867770

**Published:** 2022-04-25

**Authors:** Ruifu Yang, Bipeng Lai, Kang Liao, Baomo Liu, Lixia Huang, Shaoli Li, Jincui Gu, Ziying Lin, Yili Chen, Shuaishuai Wang, Yanli Qiu, Jiating Deng, Simin Chen, Chao Zhuo, Yanbin Zhou

**Affiliations:** ^1^Department of Pulmonary and Critical Care Medicine, The First Affiliated Hospital of Sun Yat-sen University, Guangzhou, China; ^2^Department of Clinical Laboratory, The First Affiliated Hospital of Sun Yat-sen University, Guangzhou, China; ^3^State Key Laboratory of Respiratory Disease, The First Affiliated Hospital of Guangzhou Medical University, Guangzhou, China

**Keywords:** *Acinetobacter baumannii*, *BIT33_RS14560*, biofilm, overexpression, RNA sequencing, extensively drug-resistant

## Abstract

*Acinetobacter baumannii*, a strictly aerobic, non-lactose fermented Gram-negative bacteria, is one of the important pathogens of nosocomial infection. Major facilitator superfamily (MFS) transporter membrane proteins are a class of proteins that widely exists in microbial genomes and have been revealed to be related to biofilm formation in a variety of microorganisms. However, as one of the MFS transporter membrane proteins, little is known about the role of BIT33_RS14560 in *A. baumannii*. To explore the effects of BIT33_RS14560 on biofilm formation of *A. baumannii*, the biofilm formation abilities of 62 isolates were firstly investigated and compared with their transcript levels of *BIT33_RS14560*. Then, this specific gene was over-expressed in a standard *A. baumannii* strain (ATCC 19606) and two isolates of extensively drug-resistant *A. baumannii* (XDR-Ab). Bacterial virulence was observed using a *Galleria mellonella* infection model. High-throughput transcriptome sequencing (RNA seq) was performed on ATCC 19606 over-expressed strain and its corresponding empty plasmid control strain. *Spearman’s* correlation analysis indicated a significant negative correlation (*R = −0.569*, *p = 0.000*) between the △CT levels of BIT33_RS1456 and biofilm grading of *A. baumannii* isolates. The amount of *A. baumannii* biofilm was relatively high within 12–48 h. Regardless of standard or clinical strains; the biofilm biomass in the *BIT33_RS14560* overexpression group was significantly higher than that in the control group (* p < 0.0001*). Kaplan–Meier survival curve analysis showed that the mortality of *G. mellonella* was significantly higher when infected with the *BIT33_RS14560* overexpression strain (*χ^2^ = 8.462, p = 0.004*). RNA-Seq showed that the mRNA expression levels of three genes annotated as OprD family outer membrane porin, glycosyltransferase family 39 protein, and glycosyltransferase family 2 protein, which were related to bacterial adhesion, biofilm formation, and virulence, were significantly upregulated when *BIT33_RS14560* was over-expressed. Our findings provided new insights in identifying potential drug targets for the inhibition of biofilm formation. We also developed a practical method to construct an over-expressed vector that can stably replicate in XDR-Ab isolates.

## Introduction

*Acinetobacter baumannii*, a Gram-negative bacterium that exists widely in nature, is one of the most important opportunistic pathogens responsible for nosocomial infection. Immunocompromised or severe patients in the intensive care unit are more vulnerable to this pathogen, causing a wide range of infectious diseases, including pneumonia, meningitis, peritonitis, infections of the urinary tract and skin, and posing a great threat to severe patients (Ayoub Moubareck and Hammoudi Halat, 2020). The treatment of *A. baumannii* infection has always been a challenging problem in clinical practice due to its increased resistance to commonly-used antibacterial drugs, leading to a global epidemic of multidrug-resistant *A. baumannii* (MDR-Ab; Ibrahim et al., 2021) and extensively drug-resistant *A. baumannii* (XDR-Ab; Kengkla et al., 2018).

In addition to β-lactamase and aminoglycoside modification enzymes production, active efflux, modification of target sites, and changes in outer membrane permeability (Lee et al., 2017), the drug resistance mechanism of *A. baumannii* was also found to be closely related to the formation of biofilms (Yang et al., 2019; Saipriya et al., 2020). Biofilm refers to the complex, sessile communities of microbes found either attached to a surface or buried firmly in an extracellular matrix as aggregates (Roy et al., 2018). The biofilm formation of *A. baumannii* is a complex process regulated by many factors, including the pili assembly system (Tomaras et al., 2003), BfmRS two-component regulatory systems (Gaddy and Actis, 2009), bacterial quorum-sensing system (Zhang et al., 2020), biofilm-related proteins (Bap; Fattahian et al., 2011) and outer membrane protein A (OmpA; Gaddy et al., 2009), and so on. Most clinically isolated *A. baumannii* strains have been observed to have a strong ability to form biofilms, which results in significantly low sensitivity to antimicrobial agents.

Major facilitator superfamily (MFS), a class of transporter membrane proteins, belongs to the efflux pump families and has a strong substrate specificity, which is reflected in the transportation of secondary metabolites by an ion concentration gradient. Some MFS superfamily members have been confirmed to be closely related to biofilm formation and drug resistance. For example, in a pathogenic strain *A. baumannii* AIIMS 7, *pmt*, a putative MFS transporter-like ORF of 453 bp, was identified and found to be associated with adherence, biofilm formation, and probable extracellular DNA release (Sahu et al., 2012). The efflux gene *A1S_1117*, an MFS superfamily vanillate transporter, was reported to be expressed only in biofilm cells but inhibited in planktonic cells (Rumbo-Feal et al., 2013). AbaF, another member of the MFS superfamily, was proved to mediate the efflux of intracellular fosfomycin and promote the ability to form biofilms in *A. baumannii* (Sharma et al., 2017). Therefore, the in-depth study of MFS family members is of great significance to elucidate the mechanisms of biofilm formation in *A. baumannii* and control chronic infections.

Here, we focus on a new gene, which is labeled with a locus tag of *BIT33_RS14560* in National Center of Biotechnology Information (NCBI) and presumed as a member of the MFS family. In this work, we successfully constructed two recombinant plasmids of *BIT33_RS14560* over-expression, with one aimed at the standard *A. baumannii* strain (ATCC 19606) and the other on account of two XDR-Ab isolates. We transformed them into the target strains and managed to overexpress this target gene. We found that when *BIT33_RS14560* was over-expressed, the abilities to form biofilm were significantly promoted in both standard and clinical strains of *A. baumannii*, which was also confirmed by quantitative real-time PCR (qRT-PCR) assays of 62 clinical isolates. Compared with wild-type ATCC 19606 strain, *BIT33_RS14560* overexpression strain displayed higher mortality of *Galleria mellonella* (*χ^2^ = 8.462, p = 0.004*). We also presented RNA sequencing evidence to elucidate possible mechanisms for these phenotypic changes.

## Materials and Methods

### Construction of Phylogenetic Trees

DNA or amino acid sequences of *BIT33_RS14560* gene or protein (corresponding Accession Number: CP058289.1 or WP_002047564.1) and its homologs were searched using BLAST at the (NCBI) website^1^ and downloaded in a separate FASTA format. These sequences were analyzed to construct phylogenetic trees to determine the phylogenetic relationships between the selected strains (Supplementary Tables S1, S2). The best DNA or protein model (GTR + G + I) with the highest parameter was determined and chosen before the construction of phylogenetic trees. Dendrograms were generated by Maximum Likelihood (ML) with bootstrap values corresponding to 1,000 replications using the (MEGA) 11.0 software (Tamura et al., 2021).

### Strains, Plasmids, Reagents, and Culture Media

Bacterial strains and plasmids used in this study are listed in Table 1. Of 62 *A. baumannii* isolates, 43 strains (69.4%) including two XDR-Ab isolates, XAb53 (Strain No. A53; Stored on June 2, 2021) and XAb50 (Strain No. A50; Stored on May 19th, 2020), were isolated from the First Affiliated Hospital of Sun Yat-sen University, the other 19 strains (30.6%) were obtained from the Microbiology Laboratory of State Key Laboratory of Respiratory Diseases, Guangzhou Medical University. Sources and proportion of 62 *A. baumanii* isolates used in this study were presented in Supplementary Table S3. *Escherichia coli* DH5α competent cells (Cat. No. 9057; TaKaRa) were purchased from Guangzhou Ruizhen Biotechnology Co., Ltd. The suicide plasmid pMo130-telR (Cat. No. P4951-ea; Wonder Biotech) was purchased from Guangzhou Qunlan Biotechnology Co., Ltd. Bacterial culturing was performed using Luria-Bertani (LB) agar medium (Cat. No. CM0337B; OXOID) or LB broth medium (Cat. No. CM0405; OXOID).

**Table 1:** Strains and plasmids used in this study.

Strain/Plasmid	Description	Source or Reference
Strain		
DH5α competent cell	A strain for screening or storing (recombinant) plasmids	Purchased from TAKARA
DH5α/pWH1266	A strain with a pWH1266 plasmid	Lab stock
ATCC 19606	A standard *A. baumannii* strain, wild-type	Lab stock
19,606/p-RS	An overexpressed strain of *BIT33_RS14560*, containing a pWH1266 plasmid whose TetR was deleted and replaced by *BIT33_RS14560*	This study
19,606/p	A control strain, containing a pWH1266△(TetR) plasmid but without the insertion by *BIT33_RS14560*	This study
XAb53	An XDR-Ab isolate, wild-type	This study
XAb53/p-telRs-RS	An overexpressed XDR-Ab strain of *BIT33_RS14560*, containing a pWH1266△(TetR) plasmid inserted by *BIT33_RS14560* and its AmpR was replaced by TelR	This study
XAb53/p-telRs	A control strain, containing a pWH1266△(TetR) plasmid without the insertion by *BIT33_RS14560*, but its AmpR was also replaced by TelR	This study
XAb50	An XDR-Ab isolate, wild-type	This study
XAb50/p-telRs-RS	An overexpressed XDR-Ab strain of *BIT33_RS14560*, containing a pWH1266△(TetR) plasmid inserted by *BIT33_RS14560* and its AmpR was replaced by TelR	This study
XAb50/p-telRs	A control strain, containing a pWH1266△(TetR) plasmid without the insertion by *BIT33_RS14560*, but its AmpR was also replaced by TelR	This study
Plasmid		
pWH1266	A shuttle plasmid with AmpR and TetR	Extracted from DH5α/pWH1266 strain
pMo130-telR	A suiside plasmid with TelR, NeoR, and/or KanR	Purchased from Wonder Biotech

XDR-Ab isolates refer to clinic isolates with resistance to at least one agent in all but two or fewer antimicrobial categories (Magiorakos et al., 2012). The two XDR-Ab isolates were identified by antibiotic susceptibility testing. TetR, tetracycline resistance; XDR-Ab, extensively drug-resistant *A. baumannii*; AmpR, ampicillin resistance; TelR, tellurite resistance; NeoR, neomycin resistance; and KanR, kanamycin resistance.

### Reverse Transcription PCR and qRT-PCR

Overnight culture of *A. baumannii* strain was inoculated into fresh LB broth until the mid-log phase (OD = 0.6–0.8) was reached. Total RNA was extracted using the E.Z.N.A.® Bacterial RNA Kit (Cat. No. R6950-01; Omega). The quality and concentration of RNA were determined *via* spectrophotometry (NanoDrop 2000c; Fisher Scientific, United States). For reverse transcription PCR (RT-PCR), reverse transcription was performed using ProFlex PCR System cycler (Applied Biosystems, United States). The purified RNA (1 μg/ml) was reverse-transcribed into cDNA by the Evo M-MLV RT Premix for qPCR kit (Cat. No. AG11706; Accurate Biology), in a 20 μl reaction mixture containing 4 μl of Evo M-MLVRT Master Mix and 15 μl of RNAase free water, which was incubated for 15 min at 37°C and 5 s at 85°C. Then, the qRT-PCR assay was performed on a Roche LightCycler® 960 real-time PCR system. Here, SYBR Green Premix Pro Taq HS qPCR kit (Cat. No. 78 AG11702; Accurate Biology) was employed. Around 2 μl of the resulting cDNA products were put into a 20 μl reaction mixture containing 10 μl of SYBR® Green Pro Taq HS Premix II, 0.8 μl (or 10 μM) of each primer and 6.4 μl RNase free water. The parameters of PCR were set as followed: initial heat activation at 95°C for 30 s, followed by 35 cycles of the two-step cycling condition of denaturation at 95°C for 10 s and then annealing at 60°C for 30 s. The melting data of PCR products were measured and collected following the completion of PCR cycling. The *rpoB* gene was used as an internal reference for normalization. △CT value, defined as the CT value of the target gene minus that of the reference *rpoB*, was used to calculate the *BIT33_RS14560* mRNA levels of *A. baumannii*. For each sample, three technical replicates were performed and the gene transcript levels were determined using the 2^-△△Ct^ method. Primers used in this part are listed in Table 2.

**Table 2:** Primers used in this study.

Primer	Sequences (5′–3′)	Usage
RS14560-EcoRI-F	CGC**GAATTC**GGAAATCCTTTGATTTGTGC	The amplification of *BIT33_RS14560*
RS14560-6his-BamHI-R	CGC**GGATCC**CTAATGGTGATGGTGATGAT GAGGTGCTGTTTTAAGTGAGA	
Pwh1266-F	GCCCTTTCGTCTTCAAGA	Verification of recombinant vector
Pwh1266-R	GTGATGTCGGCGATATAGG	
telR-PstI-F	TAT**CTGCAG**TTGACTTAGTTGGTATT	The amplification of the TelR cassette
telR-PvuI-R	CGC**CGATCG**TTTGAAGCTGATGTGCT	
telR-F	ACTTTATCCGCCTCCAT	Verification of the TelR cassette replacement
telR-R	GCCTTCCTGTTTTTGCT	
*rpoB*-F	GTGCTGACTTGACGCGTGAT	Amplification of the reference gene *rpoB* by fluorescence quantitative PCR
*rpoB*-R	AGCGTTCAGAAGAGAAGAACAAGTT	
RS14560-F	GTCTACAGGTTATGGCATTGG	Amplification of the target gene *BIT33_RS14560* by fluorescence quantitative PCR
RS14560-R	GCACTAATCAGCAACATCACT	

The restriction enzyme sites are highlighted using bold fonts and the His(6)-Tag is underlined in the primer sequences.

### Construction of Gene Overexpression Strains

All primers used in this study are listed in Table 2. PCR amplification was achieved using DNA polymerase (Cat. No. AG12202; Accurate 79 Biology). A plasmid DNA extraction kit (Cat. No. D2156-01; Omega), a DNA fragment purification kit (Cat. No. 9761; 77 TaKaRa), and a DNA Ligation kit (Cat. No. 6023; Accurate Biology) were also employed. The novel *BIT33_RS14560* gene was PCR-amplified from the genomes of *A. baumannii* ATCC 19606 using specific primers RS14560-EcoRI-F and RS14560-6his-BamHI-R with the following conditions—initial hold at 94°C for 4 min, followed by 32 cycles of 98°C for 10 s, 55°C for 5 s, 72°C for 8 s, and final extension at 72°C for 7 min. Purified PCR products and pWH1266 plasmid were digested with BamHI and EcoRI, then, according to the manufacturer’s instructions, ligated to make a recombinant plasmid of overexpression, named as p-RS14560. After digesting with EcoRI and BamHI, blunt ends to the large fragments of pWH1266 plasmid were created with high-fidelity DNA polymerase and then self-ligated to make into an empty plasmid, known as p. Each plasmid was transformed into *E. coli* DH5α competent cells and then introduced into the *A. baumannii* ATCC 19606 by electroporation (1.75 kV). After overnight culture (16–18 h) at 37°C, monoclonal colony PCR was performed with Pwh1266-F and Pwh1266-R primers to select positive transformants on LB agar supplemented with 50 μg/ml Carbenicillin.

For the overexpression of XDR-Ab, based on the above work, we reconstructed a specific recombinant plasmid by replacing the Ampicillin resistance (AmpR) cassette in the p-RS plasmid with the Tellurite resistance (TelR) cassette. Briefly, the TelR cassette was amplified from the plasmid pMo130-telR using telR-PstI-F/telR-PvuI-R primers. After purification, these PCR products and p-RS plasmid were digested with PstI and PvuI then ligated to make a new plasmid p-telR-RS14560. Similarly, a corresponding empty plasmid p-telR was constructed according to the above-mentioned method. Each plasmid was transformed into *E. coli* DH5α competent cells and then electroporated into *A. baumannii* XAb53 and XAb50 isolates at the same voltage of 1.75 kV. Of particular note, when selecting positive transformants with telR-F/telR-R primers, the concentration of tellurite added into the LB agar should not be completely fixed and rigid, that is, 25 μg/ml for *E. coli* DH5α and 80 μg/ml for XAb53 or XAb50 isolates. Of note, these two XDRAB clinical isolates were randomly selected according to their results of multilocus sequence typing, that was, XAb50 was assigned to ST1145 (*gltA*-1, *gyrB*-3, *gdhB*-3, *recA*-102, *cpn60*-2, *gpi*-97, and *rpoD*-3), an ST recently prevalent at the First Affiliated Hospital of Sun Yat-sen University, while XAb53 was considered a new ST (*gltA*-1, *gyrB*-3, *gdhB*-3, *recA*-102, *cpn60*-1, *gpi*-103, and *rpoD*-26). The media should be cultured overnight for 24–30 h at 37°C.

### Microtiter-Plate Test

We performed a microtiter-plate test to evaluate the biofilm formation ability of *A. baumannii* according to the method described previously (Rodríguez-Baño et al., 2008), but with certain modifications. Prior to inoculation, all strains were transferred from the stock cultures onto LB agar plates and incubated aerobically at 37°C for 18 h. Colonies from these plates were suspended in phosphate-buffered saline (PBS). After a 0.5 McFarland turbidity standard suspension (corresponding to 1.5 × 10^8^ CFU/ml) of each isolate was determined, a 100-fold dilution with sterile LB broth was prepared. Then, 200 μl of each bacterial suspension were filled into no less than six wells of a sterile 96-well flat-bottomed plastic tissue culture plate with a lid. Negative and positive control wells contained sterile LB broth and standardized suspension of *P. aeruginosa* strain ATCC 27853, respectively. The plates were covered and incubated aerobically at 37°C for 0, 6, 12, 18, 24, 30, 36, 42, 48, 60, and 72 h, respectively. Then, the content of each well was gently aspirated, and each well was washed three times with 250 ml of ultrapure deionized water and left to dry. Each well was stained for 20 min at room temperature with 200 μl of 10 g/L crystal violet. Excess stain was rinsed off by sterile water. After the plates were air-dried, each well was added with 200 μl of 33.3% glacial acetic acid and resolubilized for 20 min. Absorbance at 570 nm (OD570) of each well was measured by Epoch 2 microplate reader (BioTek). A cut-off OD (ODc) was defined as three standard deviations (SDs) above the mean OD of the negative control. All strains were classified into the following categories (Stepanović et al., 2007): non-adherent (OD ≤ ODc, 0), weakly adherent (ODc < OD ≤ 2 × ODc, +), moderately adherent (2 × ODc < OD ≤ 4 × ODc, ++), or strongly adherent (4 × ODc < OD, +++). The results were averaged after all tests were carried out three times.

### *G. mellonella* Infection Experiment

Bacterial cell suspensions at a concentration equivalent to a 0.5 McFarland Standard were prepared and followed by a 100-fold dilution with sterile LB broth. To make an infection model, the last instar healthy larvae with 2–3 cm long and 180–250 mg in weight were selected and randomly divided into groups with 10 individuals in each group. Each insect was carefully given an injection with 20 μl of bacterial solution (1.5 × 10^6^ CFU/ml) and put in a Petri dish of 8 cm in diameter. Infect at least 30 larvae for each strain. Then place the dishes at 37°C in an incubator and check larval mortality regularly over a 6 h period for 3 days. *A. baumannii* 5075, a strain with high virulence, was served as the positive control while PBS served as the negative. Mortality data were analyzed using the Kaplan–Meier method.

### Transcriptome Sequencing

Four single colonies, two derived from the overexpressed strain constructed by ATCC 19606, and two from the corresponding empty plasmid strain, were picked into 3 ml LB medium supplemented with 50 μg/ml Carbenicillin and inoculated, respectively. These cultures were grown for 18 h at 37°C and cells were then collected by centrifugation at 6,000 rpm for 10 min and washed three times with RNase free water. Total RNA extraction and the subsequent transcriptome sequencing were performed by Shanghai Majorbio Bio-pharm Technology Co., Ltd. After the quality analysis, clean data were obtained and analyzed on the online platform of Majorbio Cloud Platform.^2^ Basic functional annotation analysis was performed on the provided reference genome (Organism: *A. baumannii*; Genome ID: GCF_008632635.1). The sequences were then annotated with the following databases including NCBI Nr, NCBI Nt, Pfam,^3^ eggNOG^4^, SwissProt^5^, GO^6^, and KEGG.^7^ Differentially expressed genes (DEGs) were identified by DESeq2 software, Version 1.24.0.^8^ Those mRNAs with absolute value log2[Fold Change (FC)] ≥ 1 and *p-adjust < 0.05* were considered as significant.

### Statistical Analysis

The IBM SPSS 26.0 statistical software was used for statistical analysis. The *t* test was applied to compare the difference of measurement data, which were presented as mean ± SD. *Spearman’s* rank correlation was conducted to analyze the correlations between biofilm formation and gene expression levels. The Kaplan–Meier method was used to construct a survival curve, with a calculation of *p* value by the log-rank test. *p < 0.05* was considered to be statistically significant.

## Results

### Phylogenetic Analysis

In the case of the DNA sequences, *BIT33_RS14560* gene widely existed in all 14 *A. baumannii* strains with different identities and two clades were identified in the phylogenetic tree (Supplementary Figure S1). In the monophyletic clade I, the *BIT33_RS14560* gene was found to most closely related to *A. baumannii* ATCC 17961 and they both clustered into a subgroup with other five *A. baumannii* isolates. Interestingly, the pattern strain *A. baumannii* ATCC 17978 was subgrouped with another six isolates into clade II, indicating a distant relationship with *A. baumannii* ATCC 19606.

When it came to the amino acid sequences, the phylogenetic tree showed that the BIT33_RS14560 membrane transporter was most closely related to *A. baumannii* WP_0657191181 (Supplementary Figure S2). In addition, this protein of interest was clustered into a subgroup with its homologous proteins *A. baumannii* HAV5588984.1, *A. baumannii* HAV6132876.1, *A. baumannii* HAV4460275.1, and *Klebsiella pneumoniae* SSW87290.1. What is more, the target protein was constituted into an evolutionary clade together with *Acinetobacter nosocomialis* WP_004709884.1, *A. nosocomialis* HAB71326.1, *A. nosocomialis* WP_207694075.1 as well as *A. baumannii* HAV5002142.1.

### Investigation on the Relationship Between Biofilm-Forming Capability and *BIT33_RS14560* mRNA Levels of *A. baumannii* Isolates

In this study, when biofilm biomass was measured at 72 h by crystal violet staining, 62 *A. baumannii* isolates showed a various range of OD570 from 0.09 to 2.29 nm, indicating varying abilities of biofilm formation. Among all the isolates, 9 (14.5%), 28 (45.2%), and 17 (27.4%) were strong, moderate, and weak biofilm producers, respectively, while no biofilm was observed in eight (12.9%) isolates. Interestingly, we found that these *A. baumannii* isolates also differed considerably in their mRNA levels of *BIT33_RS14560*, with the △CT ranging from 0.47 to 19.51 (Table 3). *Spearman*’s rank correlation analysis indicated that the △CT value of *BIT33_RS14560* was negatively correlated with the biofilm rank of those *A. baumannii* isolates (*R = − 0.569, p = 0.000*), which suggested that a stronger biofilm-forming ability could be followed by a relative higher the expression level of *BIT33_RS14560* (Supplementary Table S4).

**Table 3:** The correlation between the biofilm biomass of 62 *A. baumannii* and their mRNA levels of *BIT33_RS14560*.

Strain designation	OD570 (nm)	Biofilm rank	△CT
7646	2.29	+++	13.05
3416	1.65	+++	11.12
3358	0.89	++	14.29
2599_2	2.75	+++	11.44
8017	0.57	++	17.68
7334	0.51	+	17.22
4695	0.24	−	17.15
1937	0.74	++	18.35
1421	0.44	+	19.06
4530	1.02	++	18.29
8578	0.52	+	19.33
1313	0.61	++	15.73
2499	0.43	+	19.40
8679	0.67	++	18.22
9436	0.32	+	18.55
2844	0.40	+	17.50
3679	0.77	++	17.64
8076	0.33	+	18.04
4108	0.32	+	17.17
6860	0.93	++	15.71
8036	0.28	+	18.18
5629	0.27	+	17.30
2194	0.87	++	17.14
2410	0.34	+	17.65
2241	0.93	++	17.05
1916	0.41	+	17.55
3542	0.71	++	18.66
3328	0.81	++	16.41
3508	0.42	+	19.51
3475	0.91	++	17.02
1705	0.68	++	18.48
1594	1.25	+++	17.05
1583	0.62	++	18.36
1076	1.59	+++	8.53
8124	1.42	+++	16.41
2326	1.33	+++	1.13
9252	2.16	+++	0.47
1666	0.71	++	18.90
1883	0.48	+	19.39
2346	1.05	+++	16.52
2281	0.88	++	18.47
2579	0.87	++	18.37
3388	0.83	++	18.05
1725	0.51	+	18.08
1983	0.63	++	17.31
2140	0.85	++	13.85
27	0.85	++	16.34
22	0.58	++	16.66
50	0.74	++	18.62
1	0.29	+	17.46
33	0.49	+	17.84
32	1.02	++	17.79
36	0.77	++	17.40
41	0.52	++	17.04
A67	0.13	−	18.11
A59	0.22	−	18.50
A50^▲^	0.14	−	18.01
A53^▲^	0.61	++	16.78
A143	0.09	−	18.12
A132	0.13	−	18.09
A116	0.10	−	18.34
A48	0.09	−	19.29

Different abilities of biofilm formation are presented as the categories: −*, non-adherent; +, weakly adherent; ++, moderately adherent; +++, strongly adherent.* △CT is defined as the CT value of the target gene minus that of the reference rpoB gene. ^▲^A50 referred to XAb50 while A53 referred to XAb53.

### Construction of Overexpression Vectors

**Figure 1:**
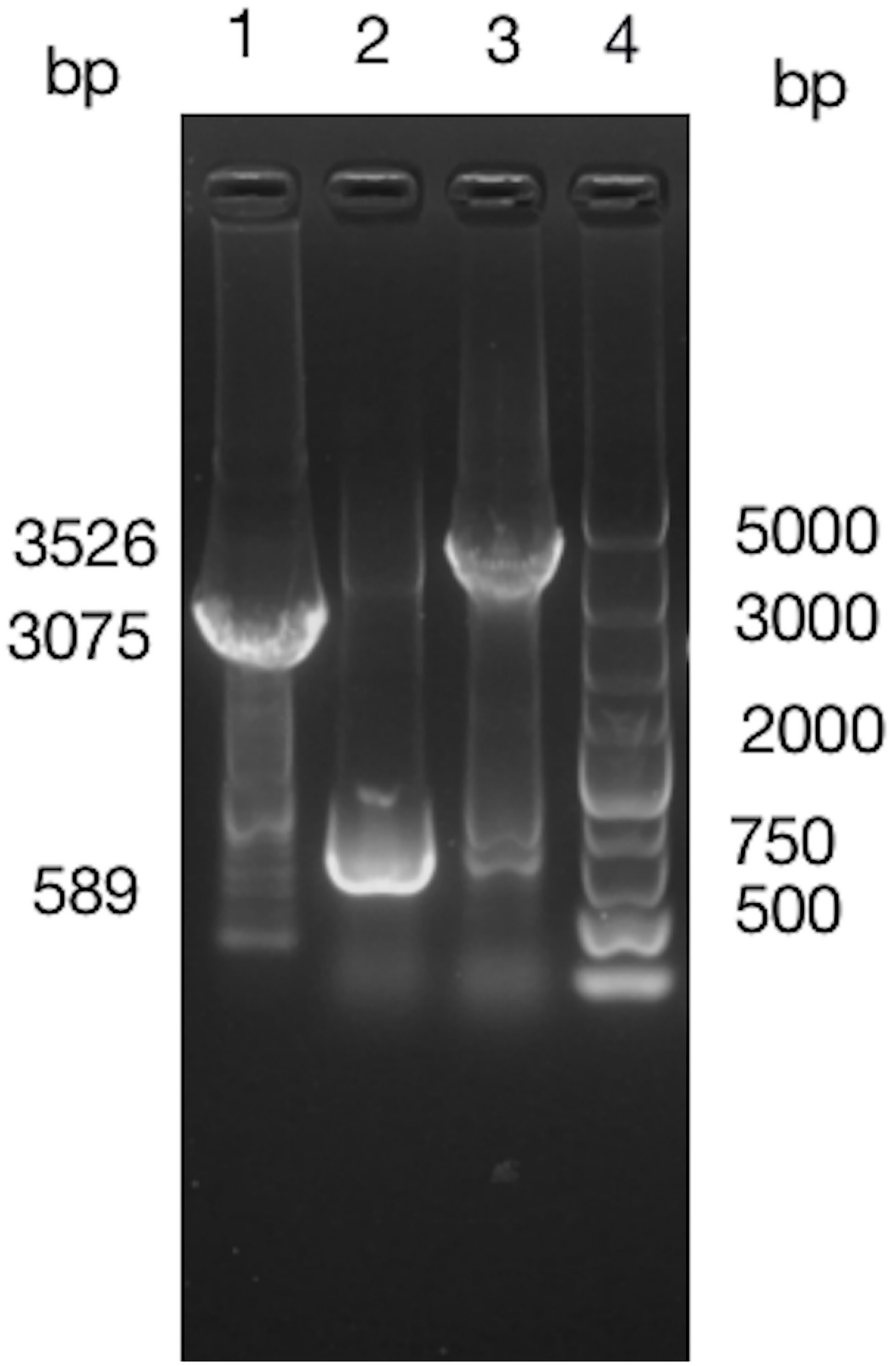
Around 1% agarose gel electrophoresis of PCR products indicates a successful construction of p-telR-RS14560 vector. The AmpR fragment in the p-RS14560 plasmid is replaced by telR cassette amplified from the pMo130telR vector. Lane 1: PCR products of telR cassette; lane 2: PCR products of p-RS14560 plasmid as negative control, lane 3: PCR products of positive clone, and lane 4: DNA marker 5,000 bp.

Fragment of *BIT33_RS14560* coding sequence (CDS) plus its preceding promoter was cloned from the ATCC 19606 genome and 1% agarose gel electrophoresis of the PCR products yielded an expected band, 1,632 bp in size. The purified PCR products were inserted into pWH1266 to construct a vector p-RS14560 for the overexpression of *BIT33_RS14560* in ATCC 19606. PCR analysis of the cloned plasmid followed by gene sequencing showed that the target fragment was successfully and correctly cloned into pWH1266 plasmid and the cloned gene was the same as *BIT33_RS14560* (NCBI Reference Sequence: NZ_MJHA01000008.1) in the GenBank. These findings indicated that the expression vector p-RS14560 was successfully constructed (Supplementary Figures S3, S4). A 3,075 bp TelR cassette was successfully cloned from the suicide vector pMo130telR and inserted into the p-RS14560 plasmid to construct a new vector p-telR-RS14560. These were confirmed by obtaining two expected bands, 3,075 and 3,526 bp in size, respectively (Figure 1). Surprisingly, we managed to overexpress the target gene *BIT33_RS14560* in two XDR-Ab isolates with this recombinant plasmid (see this article below).

### Validation of the Effect of *BIT33_RS14560* Overexpression on the Biofilm Formation in *A. baumannii* Strains

**Figure 2:**
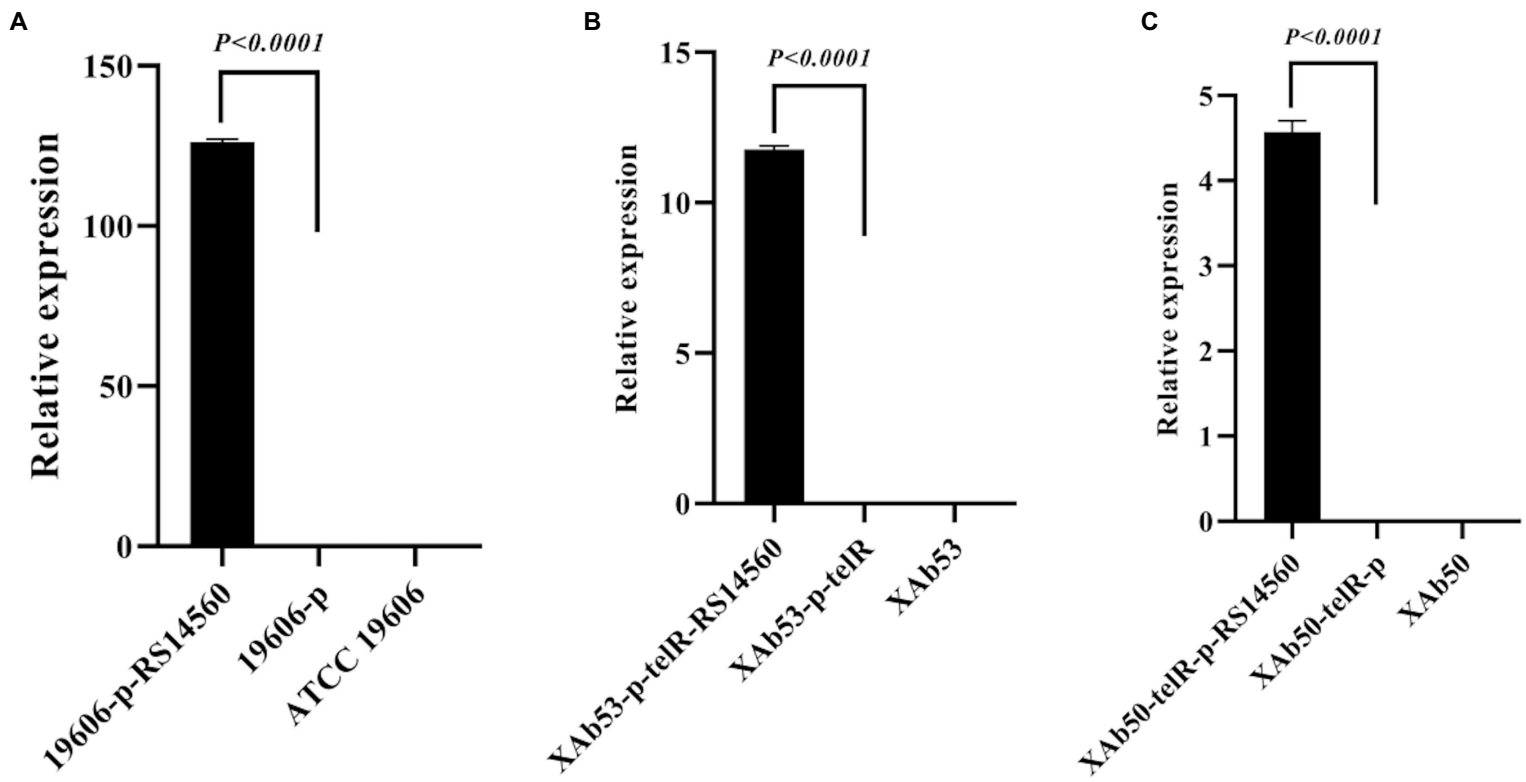
Relative expression of *BIT33_RS14560* by qRT-PCR. The *x*-axis represents the *A. baumannii* strains, and the *y*-axis represents the relative mRNA levels of *BIT33_RS14560* (2^–△CT^). *p < 0.05* indicates a statistical significance. **(A)** Overexpression of ATCC 19606 strain; **(B)** Overexpression of XAb53 isolate; and **(C)** Overexpression of XAb50 isolate.

As mentioned above, those clinical *A. baumannii* isolates with higher *BIT33_RS14560* mRNA levels tended to possess a stronger capacity of biofilm formation. To further investigate this relationship, a standard *A. baumannii* strain ATCC 19606, together with two XDR-Ab isolates, were transformed with the overexpression plasmids, p-RS14560 and p-telR-RS14560, respectively. To confirm if over-expression of *BIT33_RS14560* occurred in the ATCC 19606 and these two XDR-Ab isolates, relative quantification of the *BIT33_RS14560* transcript levels were determined by qRT-PCR with the corresponding strains transformed with empty plasmids as the control. In ATCC 19606, there was no significant difference between empty plasmid group and wild-type group, indicating that the transformation of two recombinant plasmids had little effect on the expression of *BIT33_RS14560* gene (*p > 0.05*). For ATCC 19606 strain with the plasmid overexpressing the gene of interest, when compared with its control group, the expression level of the *BIT33_RS14560* was significantly increased at 311.5 folds (*p < 0.0001*). For XAb53 strain, the relative expression levels of *BIT33_RS14560* in the overexpression group was surprisingly higher at about 586 1000-fold than that of its control group (*p < 0.0001*). Similarly, for the other XDR-Ab strain, XAb50, the overexpression group significantly displayed approximately 619 1000-fold higher *BIT33_RS14560* mRNA levels in comparison with its empty plasmid group (*p < 0.0001*). These results indicated the success of overexpression of *BIT33_RS14560* in the three *A. baumannii* strains (Figure 2). The breathtaking multiples of overexpression in two XDR-Ab isolates may probably come from their very low transcript levels (or relative high △CT values) of this target gene, as shown in Table 3.

**Figure 3:**
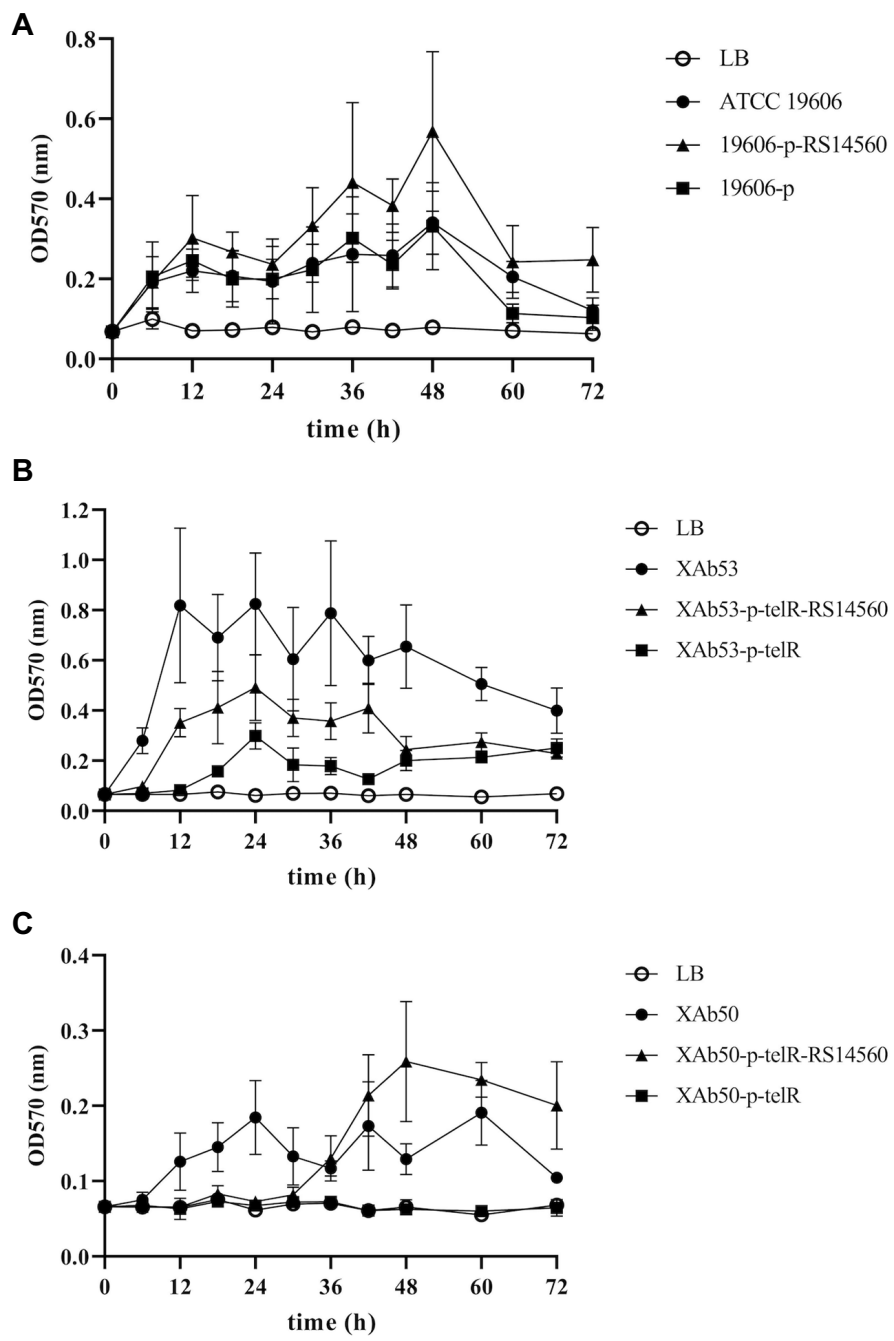
The dynamic changes of biofilm biomass produced by the nine *A. baumannii* strains at different time points. ATCC 19606, XAb53, and XAb50 are wild-type strains. 19,606-p-RS14560, XAb53-p-telR-RS14560, and XAb50-p-telR-RS14560 are *BIT33_RS14560*-overexpressed strains. 19,606-p, XAb53-p-telR, and XAb50-p-telR are empty plasmid control strains. The overexpression of *BIT33_RS14560* exhibits enhancing effects, but with various time patterns, on the biofilm formation of strains in the experimental groups. Values are means ± SD of over three independent experiments. **(A)** Wild-type and recombinant strains of ATCC 19606; **(B)** Wild-type and recombinant strains of XAb53; and **(C)** Wild-type and recombinant strains of XAb50.

To verify the effect of *BIT33_RS14560* overexpression on the biofilm formation of three *A. baumannii* strains, we evaluated the biofilm biomass with the microtiter-plate test at different points of time. We found that these three wild-type *A. baumannii* strains, including ATCC 19606 and other two XDR-Ab isolates, differed in their peak time of biofilm formation, but normally displayed relatively high levels of production within 12–48 h. When compared with each control group transformed with empty plasmids, we observed significantly higher biofilm biomass in the experimental groups of *BIT33_RS14560*-overexpression, whether in the standard strain ATCC 19606 or the two isolates XAb53 and XAb50 (Figure 3).

### *G. mellonella* Survival Influenced by *BIT33_RS14560* Overexpression

**Figure 4:**
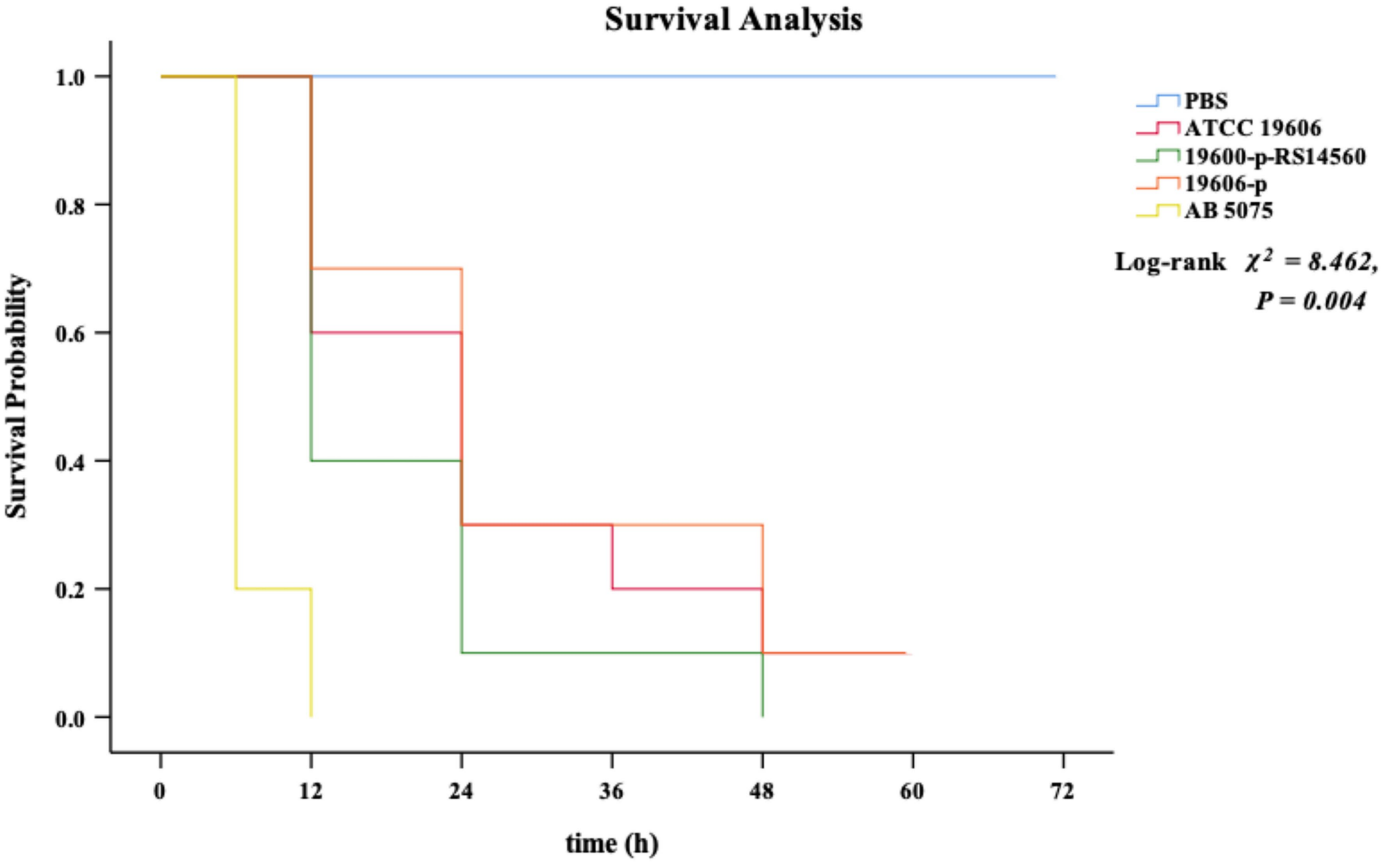
Kaplan–Meier curve analysis of the survival of *G. mellonella* after infection. PBS is the negative control group, ATCC 19606 is the wild-type strain, 19,606-p-RS14560 is the overexpression strain, 19,606-p is the empty plasmid control group, and *A. baumannii* 5075 is the positive control group. PBS, phosphate buffered saline. *p < 0.05* indicates a statistical significance.

We used the *G. mellonella* infection model to investigate the effect on the virulence of the standard strain ATCC 19606 when *BIT33_RS14560* was overexpressed. None of the *G. mellonella* died within 72 h when given with the equivalent PBS. By contrast, all *G. mellonella* died within 12 h following infection with the high-virulent strain *A. baumannii* 5075, which served as the positive control. When infected with the ATCC 19606 wild-type strain, *G. mellonella* mortality was 40.0% within 12 h and then rose to 70.0% at 24 h. In contrast, *G. mellonella* mortality following infection with the *BIT33_RS14560* overexpression strain was 60.0% at 12 h and reached 90% within 24 h. No significant difference was observed in the survival rates between the wild-type strain ATCC 19606 and its empty plasmid strain 19,606-p (*p > 0.05*). As shown in Figure 4, Kaplan–Meier survival curve analysis showed that the survival rate of *G. mellonella* was significantly lower when infected with the *BIT33_RS14560* overexpression strain (*χ^2^ = 8.462, p = 0.004*).

### Screening of the Candidate Genes Related to *A. baumannii* Biofilm Formation Influenced by Overexpressed *BIT33_RS14560* Based on RNA-Seq

**Figure 5:**
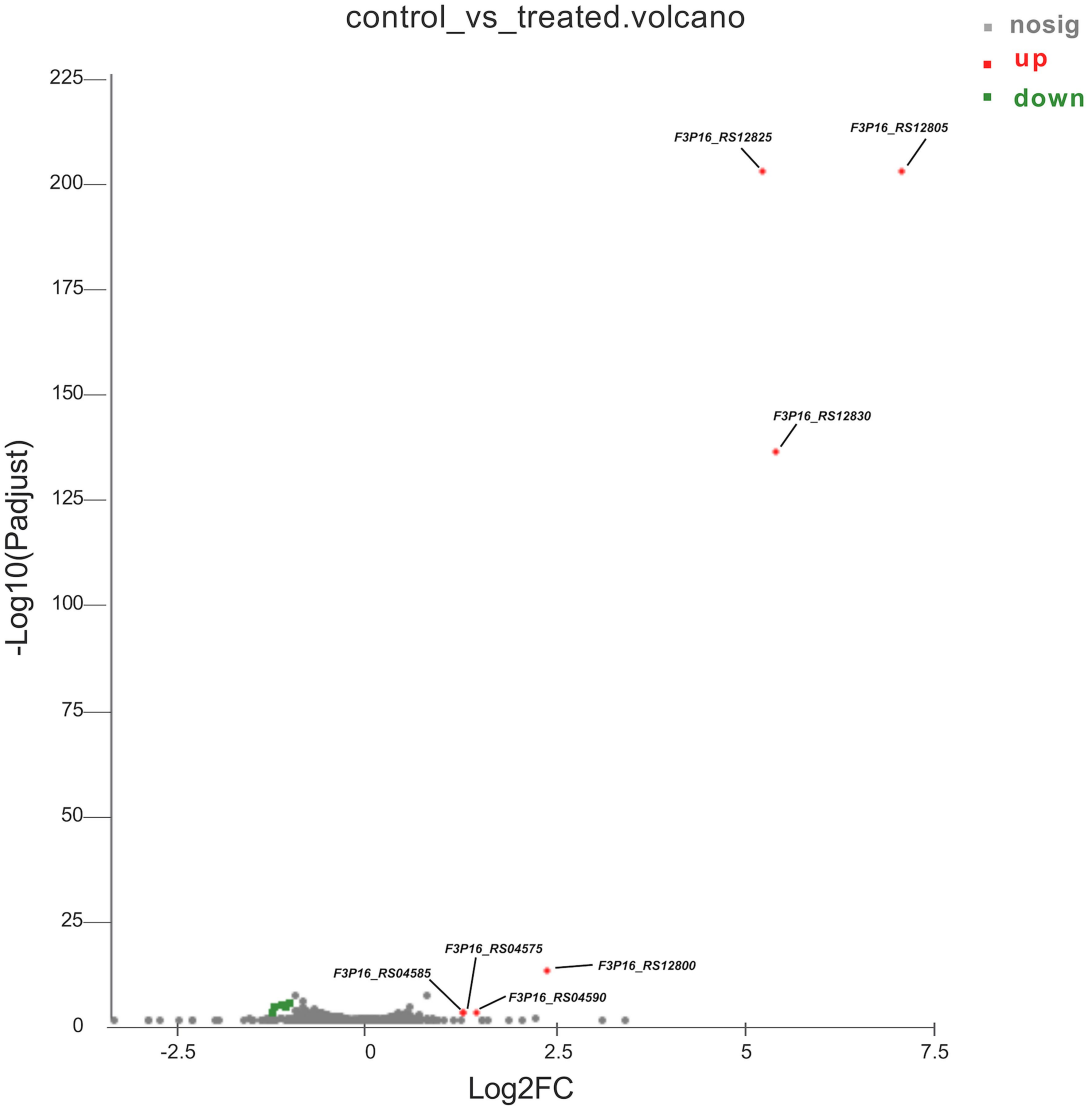
The volcano plot of differentially expressed genes identified by RNA-Seq. The *x*-axis represents the log_2_(FC), and the *y*-axis represents –log_10_ (*p-adjust*) calculated by the Student’s *t*-test. The red dots represent the upregulated genes with statistical significance (* p-adjust < 0.05* and |log_2_(FC)| ≥ 1). The green dots represent genes with down-regulated expression, while the gray ones represent genes with no statistical significance. Those upregulated DEGs are indicated. FC, fold change.

**Figure 6:**
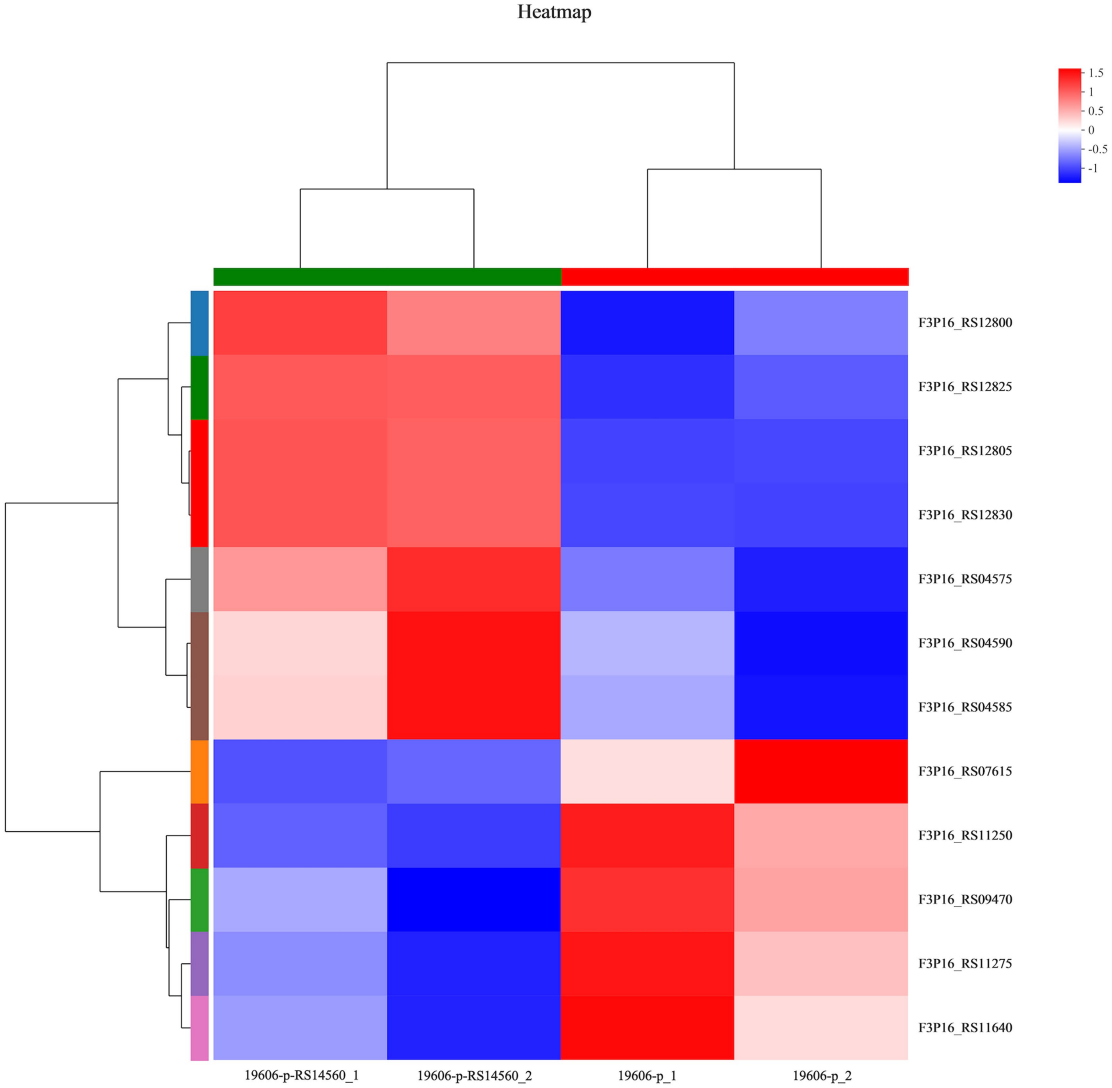
Heatmap of differentially expressed genes. 19,606-p-RS14,560_1 and 19,606-p-RS14,560_2 represent the treated groups while 19,606-p_1 and 19,606-p_2 represent the control groups. FC, fold change.

**Figure 7:**
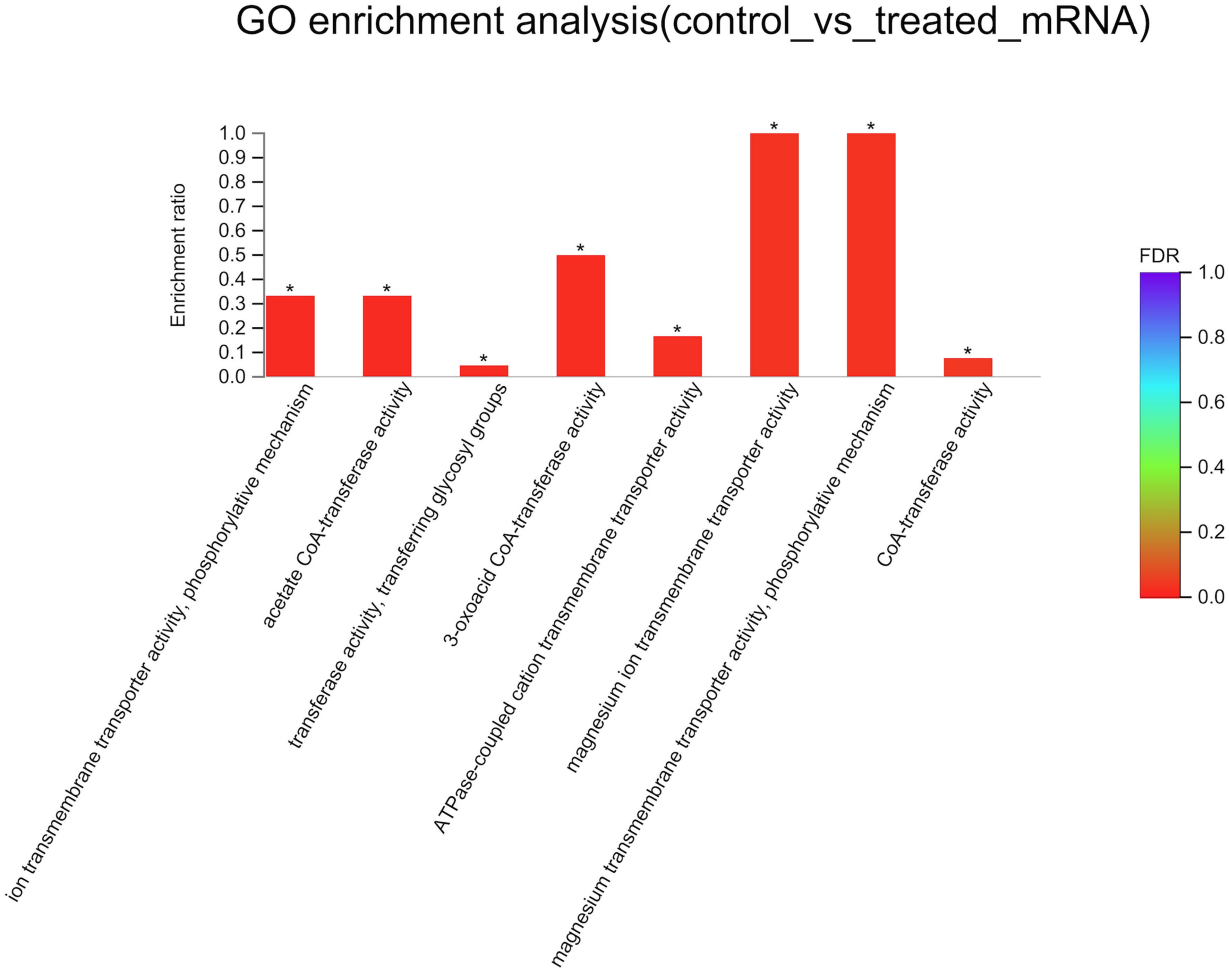
GO enrichment analysis of differentially expressed genes. The *x*-axis represents GO terms, and the *y*-axis represents the enrichment ratio. A higher ratio means a greater degree of enrichment. GO, Gene Ontology; *FDR*, false discovery rate. ^*^FDR < 0.05.

**Figure 8:**
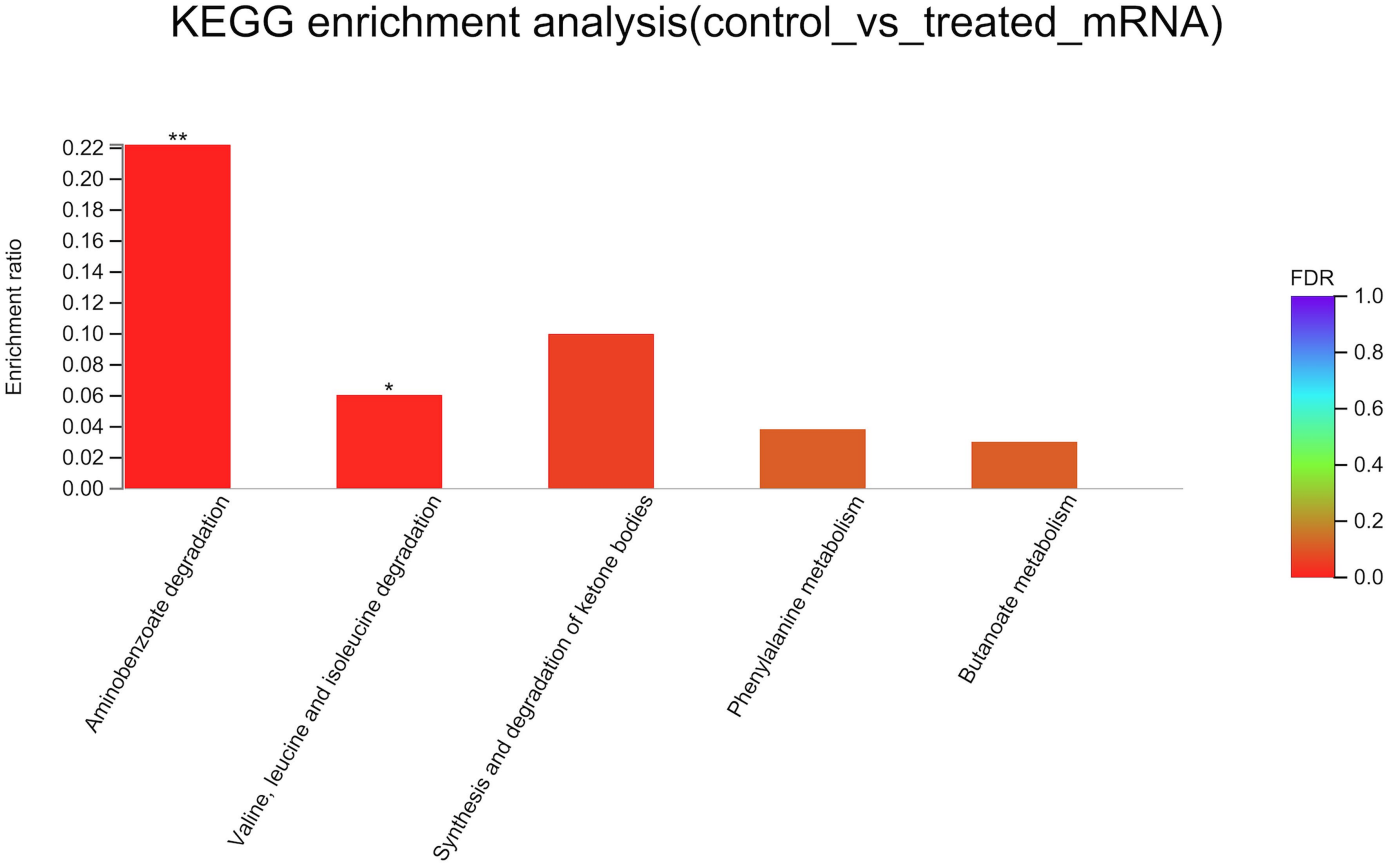
KEGG enrichment analysis of differentially expressed genes. The *x*-axis represents KEGG terms, and the *y*-axis represents the enrichment ratio. A higher ratio means a greater degree of enrichment. KEGG, Kyoto Encyclopedia of Genes and Genome. *FDR*, false discovery rate. ^*^FDR < 0.05, ^**^FDR < 0.01.

To better understand the mechanisms of the relationship between *BIT33_RS14560* overexpression and *A. baumannii* biofilm formation, in the current study, we conducted a comparative transcriptomic analysis of four samples, including the treated group (two samples derived from ATCC 19606 with target gene overexpression) and the control group (two samples derived from ATCC 19606 with empty plasmid). A total of over 7.0 Gigabyte raw data were obtained, and the percentage of Q30 (an important index for the assessment of RNA-Seq quality, a higher value usually indicates a lower probability of base error) reached above 94.65%. Totally, 3,232 expressed genes were detected in this analysis, including 3,208 mRNAs and 24 small RNAs (sRNAs). Under the threshold of |log2(FC)| ≥ 1 and *p-adjust < 0.05*, a total of 12 DEGs (seven upregulated and five downregulated) were screened for subsequent analysis. The volcano plot and heatmap of these DEGs were drawn in Figures 5, 6, respectively. To further explore the biological functions of these DEGs, we performed GO and KEGG enrichment analysis. As presented in Figure 7, none of these DEGs were involved in GO biological process or cellular component category. However, in molecular function(MF)category, they significantly enriched in “magnesium transmembrane transporter activity, phosphorylative mechanism” (GO:0015444), “3-oxoacid CoA-transferase activity” (GO:0008260), “acetate CoA-transferase activity” (GO:0008775), “ATPase-coupled cation transmembrane transporter activity” (GO:0019829), and “transferase activity, transferring glycosyl groups” (GO:0016757; all *FDR<0.05*). In terms of the KEGG pathway, “aminobenzoate degradation” (map00627) and “valine, leucine, and isoleucine degradation” (map00280) were involved (*FDR < 0.05*; Figure 8).

## Discussion

### Phylogenetic Analysis

To investigate the distribution of the *BIT33_RS134560* gene and its encoding protein in *A. baumannii* strains as well as their relationship, we conducted phylogenetic tree analysis. Our results showed that this gene existed in all the 14 *A. baumannii* strains of different genomic homology (Supplementary Figure S1). Similarly, the BIT33_RS14560 protein and its homologous ones could be found in many other strains of *A. baumannii*, even in other *Acinetobacter* spp. and *K. pneumoniae* (Supplementary Figure S2). These results indicated a wide distribution of the *BIT33_RS134560* gene and its protein in *A. baumannii* strains. It was interesting to note that this target gene in ATCC19606 was very closely related to ATCC17961, but not to ATCC17978, although they were all pattern strains of *A. baumannii*. We have not figured out why and how this gene evolved the way it did. Further study of the difference in the expression of the *BIT33_RS14560* gene between these two clades may shed light on the causes of this phenomenon.

In addition, that the BIT33_RS14560 protein was closely related to the MFS homologs of *A. nosocomiae* and *K. pneumoniae* indicated potential pathogenicity of this protein due to the fact that the latter two bacteria were also important pathogens of nosocomial infections (Nho et al., 2015; Wang et al., 2020). Therefore, we believed this gene of interest needed to be noticed.

### Construction of Overexpression Vector in XDR-Ab Isolates

The *E. coli*/*Acinetobacter* shuttle vector pWH1266 containing AmpR and TetR was initially generated for the cloning experiments of *Acinetobacter calcoaceticus* and *E. coli* (Hunger et al., 1990) and has been successfully applied in the overexpression construction of *A. baumannii* (Zander et al., 2013; Liou et al., 2014). However, for XDR-Ab, it should be mentioned that due to its extreme drug resistance, the number of antibiotic selection markers for transformants was greatly limited. One solution to this problem was that the antibiotic concentration should be increased as much as possible within the tolerant range of the vector’s resistance. In this case, we made our attempt to transform the recombinant plasmid p-RS14560, which contained only AmpR, into an XDR-Ab isolate by increasing the concentration of Carbenicillin from 50 to 1,700 μg/ml. However, we failed because the XDR-Ab was still able to grow at such a high concentration of Carbenicillin. To achieve this goal of overexpression in XDR-Ab, a replacement of AmpR by other resistant genes was the key to the deal.

When we made our attempts to knock out this target gene using a marker-less method (Amin et al., 2013), the TelR cassette of pMo130telR vector caught our attention. This suicide vector was derived from the modification of the pMo130 vector (Hamad et al., 2009) by carrying a TelR cassette (Sanchez-Romero et al., 1998). Researchers had tested *A. baumannii* isolates, including MDR-Ab isolates, and found that they were susceptible to tellurite (Amin et al., 2013). We also tested several XDR-Ab isolates to confirm their susceptibilities to tellurite and found that they could be counter-selected in LB agar containing tellurite of 30–100 μg/ml (data not shown). Fortunately, we managed to overexpress the target gene *BIT33_RS14560* in two XDR-Ab isolates by replacing the AmpR with the TelR. The transformation of two recombinant vectors, p-telR and p-telR-RS14560, might to some extent make an impact on the transcript expression of *BIT33_RS14560*. This could be explained by the fitness cost of bacteria (San Millan and MacLean, 2017; Nang et al., 2018). Large plasmids could impose a metabolic burden for host bacterial strains (Ma et al., 2018). However, to our knowledge, this was the first time to achieve stable gene overexpression in XDR-Ab by the replacement of AmpR with telR, which would provide guidance for further investigations on XDR-Ab strains.

### Biofilm-Forming Capabilities and *BIT33_RS14560* mRNA Levels of *A. baumannii* Isolates

Like many other bacteria, *A. baumannii* displays a robust biofilm formation on many abiotic surfaces in hospital environments, raising the risk of chronic infections. Previous studies have shown that the degree of biofilm formation varied considerably depending on the *A. baumannii* isolates (Rodríguez-Baño et al., 2008). Our study also showed different abilities of biofilm formation among 62 *A. baumannii* isolates when measured at 72 h. Those with robust abilities of biofilm formation were more likely to survive under desiccation and nutrition-limiting conditions, which explained why some clinical isolates possessed more tenacious vitality in hospitals than others. As shown in Supplementary Table S4, *Spearman’s* rank correlation analysis suggested that a stronger biofilm-forming ability could be followed by a relatively higher expression level of *BIT33_RS14560* (the △CT value vs. biofilm rank, *R = −0.569, p = 0.000*). This was validated by overexpression of the *BIT33_RS14560* gene in the standard strain ATCC 19606 and the two isolates XAb53 and XAb50 (Figure 3).

Here, three wild-type *A. baumannii* strains used for the construction of *BIT33-RS14560* overexpression normally displayed relatively high levels of production within 12–48 h, which was in accordance with the previous study (Rodríguez-Baño et al., 2008). However, the enhancing effects of *BIT33_RS14560* on biofilm formation appeared to be variable in these three *A. baumannii* strains (Figure 3). Within 48 h, for the ATCC 19606 strain, overexpression of *BIT33_RS14560* exhibited a gradual upward trend of facilitating biofilm formation, while in the XAb53 isolate; this effect seemed to be more stable, keeping it in fairly high levels of biofilm biomass. However, when it came to XAb50 isolate, the amount of biofilm remained relatively unchanged over the past 30 h, then, rose sharply into about 3.24-fold (data not shown) at 48 h. Further investigation is warranted to elucidate the mechanisms for this phenomenon.

### RNA-Seq Reveals Possible Mechanisms by Which *BIT33_RS14560* Affects Biofilm Formation and Virulence of *A. baumannii*

Currently, *A. baumannii* biofilm formation, of which the mechanisms are still not fully elucidated, is still a tough issue in the treatment of refractory and chronic infections. As one of the largest superfamily of secondary carriers known to date, MFS family membrane transporters exist widely in the whole biological world (Reddy et al., 2012) and they are considered to be closely related to a wide variety of life phenomena due to a basic function of assisting in transmembrane transport of certain substances, including monosaccharides, oligosaccharides, amino acids, enzyme cofactors, drugs, and so on (Lorca et al., 2007; Chen et al., 2008; Yen et al., 2010). In bacteria, MFS superfamily proteins have been found not only to play an important role in the transport of many substances but also to be a member of the efflux pumps large families (Pasqua et al., 2019), which are involved in biofilm formation and drug resistance (Bay et al., 2017; Saranathan et al., 2017; Poudyal and Sauer, 2018; Tang et al., 2020).

BIT33_RS14560, although considered to be categorized into the MFS family, is still lacking reports about its effects on the biofilm formation of *A. baumannii*. In this study, we conducted a comparative transcriptomic analysis to investigate the possible mechanisms by which *BIT33_RS14560* overexpression had an impact on the biofilm formation and virulence of *A. baumannii* ATCC19606. As shown in Table 4, we found that three DEGs, including *F3P16_RS12800*, *F3P16_RS04575*, and *F3P16_RS04585*, although displayed different up or down multiples, were hypothesized to be related more or less to bacterial biofilm formation or virulence.

**Table 4:** Differentially expressed genes identified by RNA-Seq.

Gene identity	Description	log_2_(FC)	*P*	*P-adjust*	Regulation
*F3P16_RS12805*	MFS transporter	7.100932775	0	0	up
*F3P16_RS12825*	Aromatic ring-hydroxylating dioxygenase subunit alpha	5.270041648	7.88E-206	1.26E-202	up
*F3P16_RS12830*	PDR/VanB family oxidoreductase	5.447523987	5.15E-139	5.51E-136	up
*F3P16_RS12800*	OprD family outer membrane porin	2.405050832	1.88E-15	1.50E-12	up
*F3P16_RS04590*	ChbG/HpnK family deacetylase	1.473469534	5.46E-05	0.009736754	up
*F3P16_RS04575*	Glycosyltransferase family 39 protein	1.29285042	0.000175063	0.02065165	up
*F3P16_RS04585*	Glycosyltransferase family 2 protein	1.280990985	0.000240526	0.024890574	up
*F3P16_RS07615*	Magnesium-translocating P-type ATPase	−1.011635328	2.24E-07	8.99E-05	down
*F3P16_RS09470*	CoA transferase subunit A	−1.098553066	8.82E-07	0.000314477	down
*F3P16_RS11250*	DcaP-like protein	−1.209536537	1.62E-06	0.000518977	down
*F3P16_RS11275*	Methylcrotonoyl-CoA carboxylase	−1.052143105	1.93E-06	0.000541303	down
*F3P16_RS11640*	Phenylacetate-CoA oxygenase subunit PaaJ	−1.227463257	9.57E-05	0.013948752	down

Differentially expressed genes were screened by DESeq2 software, under a threshold of |log_2_(FC)| ≥ 1 and *p*-adjust < 0.05. Of note, the F3P16_RS12805 gene refers to the target gene BIT33_RS14560. FC, fold change.

*F3P16_RS12800* was annotated as an OprD family outer membrane porin. The OprD family was described first for *P. aeruginosa*, but ever since that time, it has been found in many metabolically versatile soil bacteria and comprises over 100 members (Tamber et al., 2006). In the current study, we found that the overexpression of *BIT33_RS14560* led to a significant upregulation of *F3P16_RS12800*, suggesting that in *A. baumannii*, *BIT33_RS14560* may enhance biofilm formation by upregulating the expression of this outer membrane porin. This was supported by a previous comparative proteomics study (Cabral et al., 2011) revealing that some membrane proteins including OprD-like protein were involved in the adhesion and biofilm formation of *A. baumannii*.

Most importantly, we got *F3P16_RS04575* and *F3P16_RS04585*, both encoded proteins belonging to the glycosyltransferase (GT) family and were upregulated when *BIT33_RS14560* was overexpressed. GTs mainly participate in the transfer process of glycosyl from an activated donor to a receptor, such as sugar, lipid, protein, and nucleic acid and play an important role in pathogenic adhesion, biofilm formation, and virulence (Breton et al., 2006; Ribet and Cossart, 2010a). Structural analysis of three-dimensional (3D) protein fold has shown that the catalytic domains of GTs are categorized into GT-A, GT-B, and GT-C three types, the most common of which are GT-A and GT-B (Moremen and Haltiwanger, 2019). GT-B or GT-C is believed to play a role in adhesion, biofilm formation, and virulence of pathogenic bacteria while GT-A enters the host cell and interferes it with post-translational modifications thus affecting its signal transduction, protein translation, and immune response. It was also important to note that the protein glycosylation of GT was closely related to the virulence of pathogenic bacteria (Ribet and Cossart, 2010b; Yakovlieva and Walvoort, 2020). Many bacterial pathogens were found to bear toxic GTs that interfere with host post-translational modifications to promote their own survival and replication (Lu et al., 2015). Interestingly, when we further analyzed with the Carbohydrate-Active Enzymes (CAZy) database,^9^ we found that the proteins encoded by *F3P16_RS04575* and *F3P16_RS04585* belonged to GT-C and GT-A, respectively. This might explain why ATCC 19606 became more virulent when *BIT33_RS14560* was overexpressed (Figure 4).

It was reported that proteins and carbohydrates were the main components of biofilm formation (Costerton et al., 1995). Amino acid metabolism, glycerolipid, and tricarboxylic acid cycle were also observed to be mostly affected during bacterial biofilm formation (Lu et al., 2019). Our results of GO and KEGG analysis (Figures 7, 8) also indicated that most DEGs participated in complicated metabolic processes including amino acid, carbohydrate metabolism, which also suggested the complexity of bacterial biofilm formation. However, the specific effects of these DEGs on *A. baumannii* biofilm formation and what metabolic pathways these DEGs may be involved in remained further investigations.

## Conclusion

In this study, we demonstrated that when *BIT33_RS14560*, a member of the MFS family, was over-expressed, the abilities to form biofilm and the virulence of *A. baumannii* were significantly enhanced, no matter in standard or clinically isolated strains. This gene of interest existed widely in *A. baumannii* strains and its protein was closely related to the MFS homologs of *A. nosocomiae* and *K. pneumoniae*. Three DEGs including *F3P16_RS12800*, *F3P16_RS04575*, and *F3P16_RS04585*, annotated as OprD family outer membrane porin, glycosyltransferase family 39 protein and glycosyltransferase family 2 protein, respectively, were identified by RNA-Seq and speculated to be related to the biofilm formation and virulence of *A. baumannii*, which needs further studies. These findings provided new insights in identifying potential drug targets for the inhibition of biofilm formation. Besides, we developed a method for the construction of XDR-Ab overexpression, which may promote further microbiological research on their genotypes and phenotypes.

## Data Availability Statement

The data presented in the study are deposited in the Sequence Read Archive (SRA) repository, accession number at: https://www.ncbi.nlm.nih.gov/sra/PRJNA821219.

## Author Contributions

RY and BLa contributed to the conception and design of this study. RY, BLa, and BLi performed the experiments. KL, YC, and CZ provided the study materials. RY, LH, SL, JG, ZL, and SW analyzed and interpreted the data. YZ, CZ, and KL gave administrative supports. All authors contributed to the article and approved the submitted version.

## Funding

This work is supported by grants from the National Natural Science Foundation of China (81570008, YZ) and the Natural Science Foundation of Guangdong Province of China (2021A1515010480, YZ).

## Conflict of Interest

The authors declare that the research was conducted in the absence of any commercial or financial relationships that could be construed as a potential conflict of interest.

## Publisher’s Note

All claims expressed in this article are solely those of the authors and do not necessarily represent those of their affiliated organizations, or those of the publisher, the editors and the reviewers. Any product that may be evaluated in this article, or claim that may be made by its manufacturer, is not guaranteed or endorsed by the publisher.

## Footnotes

^1^ http://www.ncbi.nlm.nih.gov/BLAST/

^2^ www.majorbio.com

^3^ http://pfam.xfam.org/

^4^ http://eggnogdb.embl.de/

^5^ http://uniprot.org

^6^ http://www.geneontology.org/

^7^ http://www.genome.jp/kegg/

^8^ http://bioconductor.org/packages/stats/bioc/DESeq2/

^9^ http://www.cazy.org

## Supplementary Material

Supplementary Figure S1 Phylogenetic analysis of *BIT33_RS14560* gene. Bootstrap values >50 are displayed on the branches. A higher bootstrap value indicates a better reliability.

Supplementary Figure S2 Phylogenetic analysis of BIT33_RS14560 protein. Bootstrap values >50 are displayed on the branches. A higher bootstrap value indicates a better reliability.

Supplementary Figure S3 The construction of p-RS14560 overexpressed vector. **(A)** The amplification of *BIT33_RS14560* fragment. Lane 1: DNA marker 2,000 bp, lane 2: sample; **(B)** PCR products of pWH1266 plasmid as negative control. Lane 1: DNA marker 2,000 bp, lane 2: PCR products of pWH1266 plasmid as negative control **(C)** Recombinant plasmid is successively transformed into competent *E. coli* DH5α and ATCC 19606 strains, with the Pwh1266-F/Pwh1266-R primers being used to ensure the success of directional linkage. Lane 1: DNA marker 2,000 bp, lane 2: PCR products of p-RS14560 plasmid as positive control, lane 3: PCR products of positive clone.

Supplementary Figure S4 A representative part of the sequence alignment between the target gene in the p-RS14560 vector and the comparative analysis to the *BIT33_RS14560* gene.
