# Supplementary figures and images for: Overexpression of BIT33_RS14560 Enhances the Biofilm Formation and Virulence of Acinetobacter baumannii
Source: Front Microbiol. 2022 Apr 25;13:867770. doi: 10.3389/fmicb.2022.867770 (PMC9083411; doi:10.3389/fmicb.2022.867770)

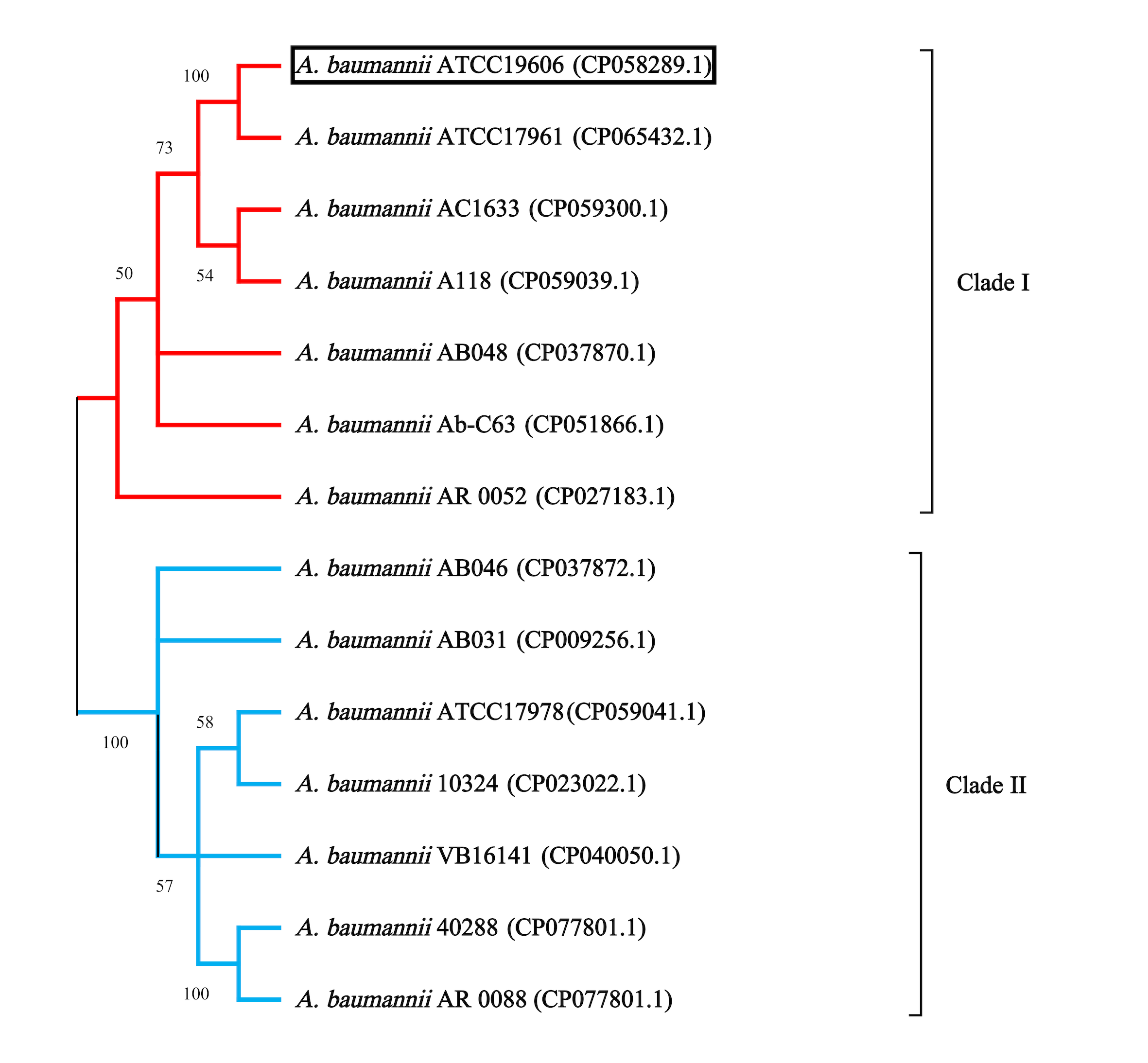

Supplement: Supplementary Figure S1 — Phylogenetic analysis of BIT33_RS14560 gene. Bootstrap values >50 are displayed on the branches. A higher bootstrap value indicates a better reliability. [file Image_1.TIF]

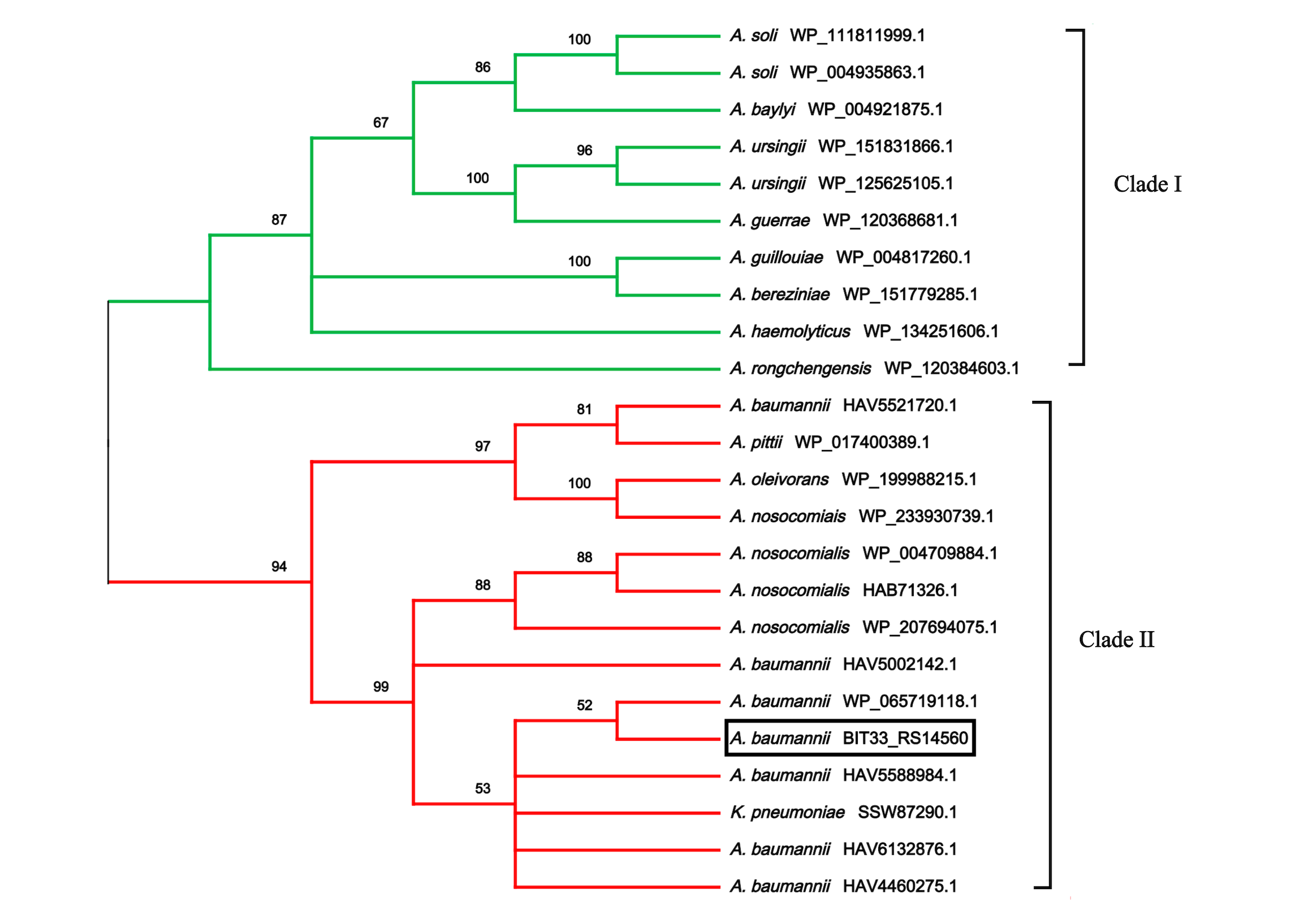

Supplement: Supplementary Figure S2 — Phylogenetic analysis of BIT33_RS14560 protein. Bootstrap values >50 are displayed on the branches. A higher bootstrap value indicates a better reliability. [file Image_2.TIF]

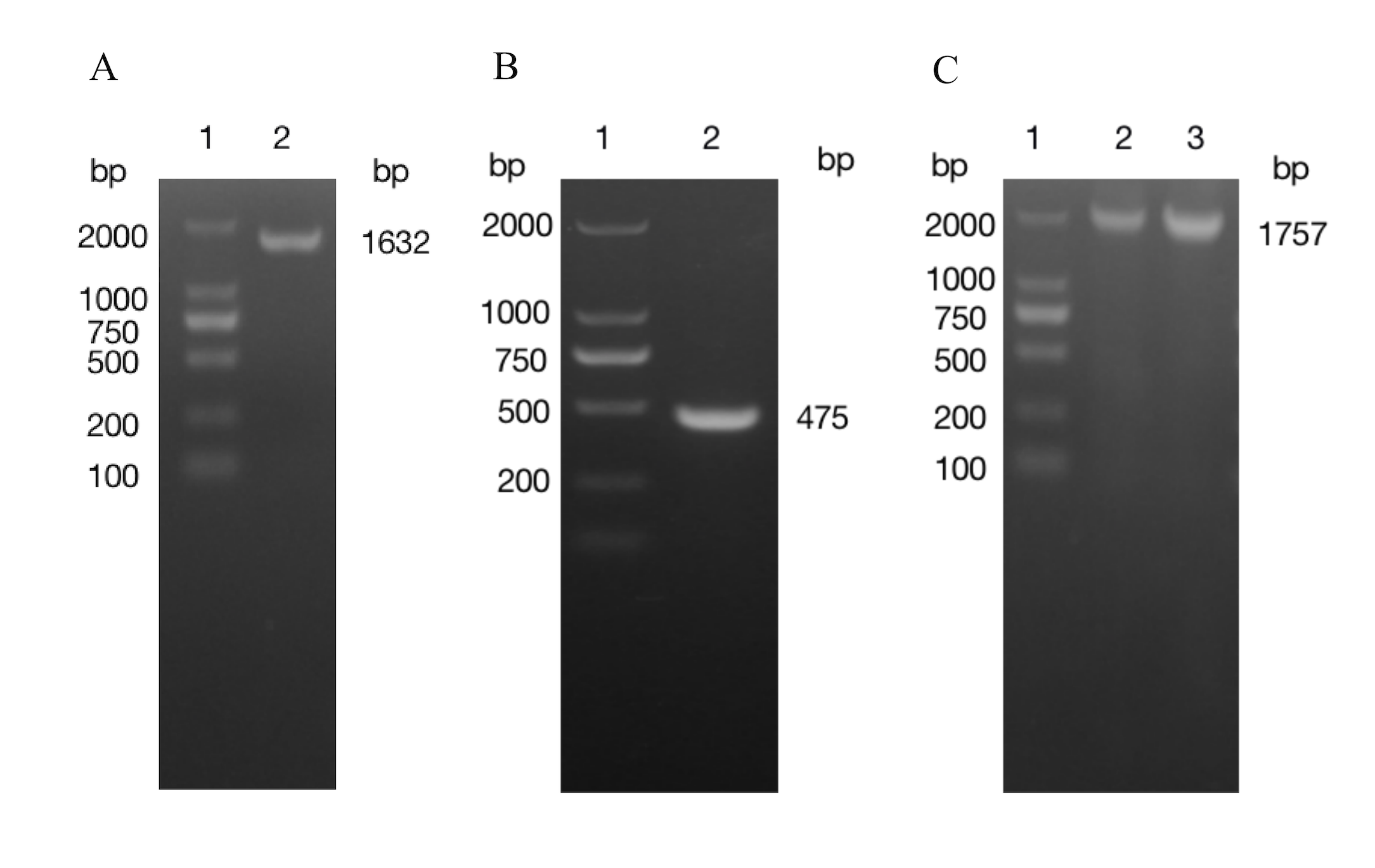

Supplement: Supplementary Figure S3 — The construction of p-RS14560 overexpressed vector. (A) The amplification of BIT33_RS14560 fragment. Lane 1: DNA marker 2,000 bp, lane 2: sample; (B) PCR products of pWH1266 plasmid as negative control. Lane 1: DNA marker 2,000 bp, lane 2: PCR products of pWH1266 plasmid as negative control (C) Recombinant plasmid is successively transformed into competent E. coli DH5α and ATCC 19606 strains, with the Pwh1266-F/Pwh1266-R primers being used to ensure the success of directional linkage. Lane 1: DNA marker 2,000 bp, lane 2: PCR products of p-RS14560 plasmid as positive control, lane 3: PCR products of positive clone. [file Image_3.TIF]

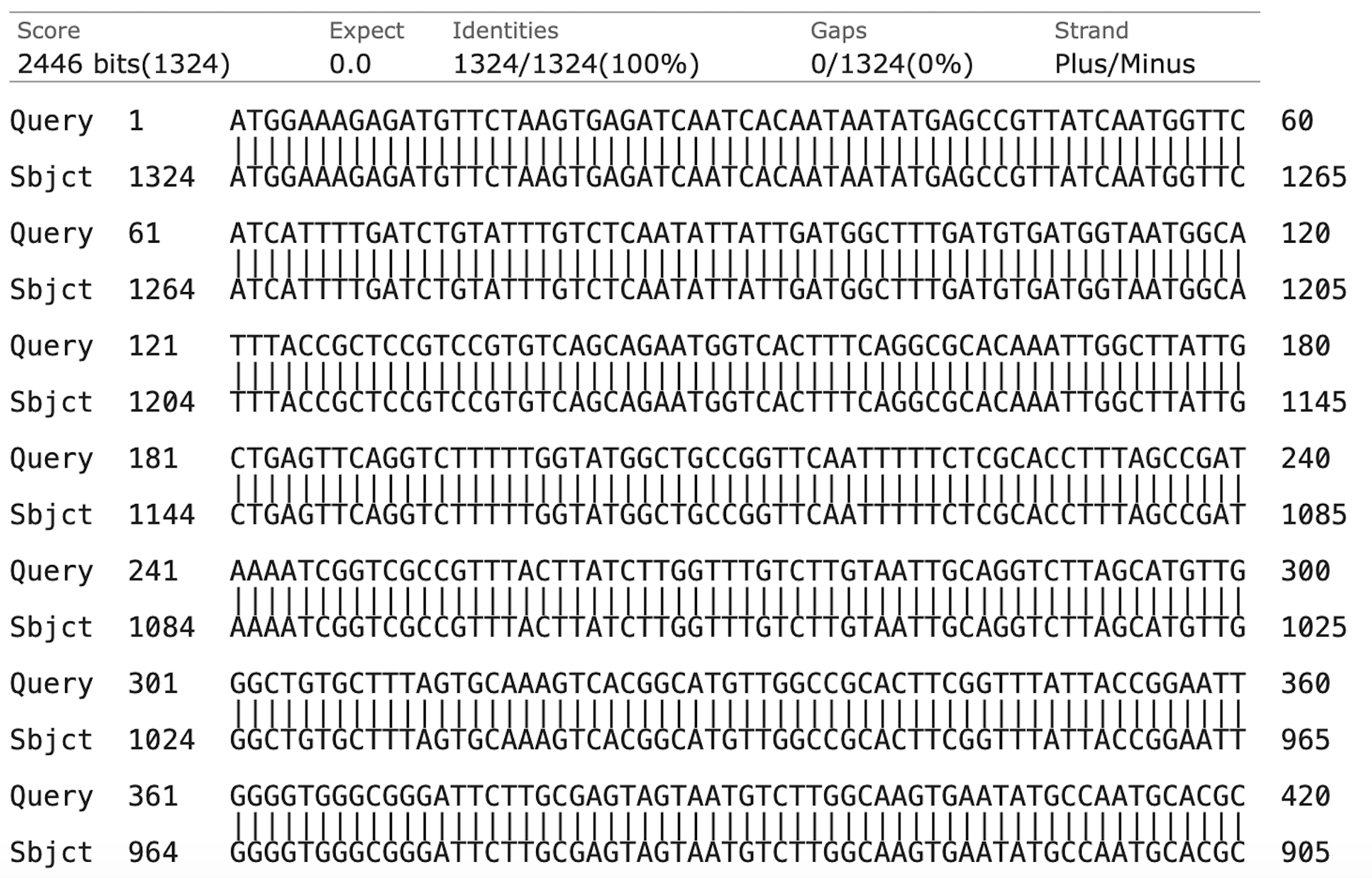

Supplement: Supplementary Figure S4 — A representative part of the sequence alignment between the target gene in the p-RS14560 vector and the comparative analysis to the BIT33_RS14560 gene. [file Image_4.TIF]
