# Supplementary material for: Overexpression of BIT33_RS14560 Enhances the Biofilm Formation and Virulence of Acinetobacter baumannii
Source: Front Microbiol. 2022 Apr 25;13:867770. doi: 10.3389/fmicb.2022.867770 (PMC9083411; doi:10.3389/fmicb.2022.867770)
Supplement: Supplementary file 5 [file Data_Sheet_1.docx]

| **Supplementary Table S1 \|** Amino acid sequences of the chosen strains used for the construction of phylogenetic trees. | | | |
| --- | --- | --- | --- |
| Strain | Accession number | Annotation | Identity(%) |
| *A. baumannii* | WP_002047564.1^▲^ | MFS transporter | 100 |
| *A. baumannii* | WP_065719118.1 | MFS transporter | 99.78 |
| *A. baumannii* | HAV4460275.1 | MFS transporter | 99.33 |
| *A. baumannii* | HAV5588984.1 | MFS transporter | 99.33 |
| *K. pneumoniae* | SSW87290.1 | 4-hydroxybenzoate  transporter | 99.21 |
| *A. nosocomialis* | WP_207694075.1 | MFS transporter | 99.11 |
| *A. baumannii* | HAV5002142.1 | MFS transporter | 98.88 |
| *A. nosocomialis* | WP_004709884.1 | MFS transporter | 98.66 |
| *A. baumannii* | HAV6132876.1 | MFS transporter | 98.44 |
| *A. nosocomialis* | HAB71326.1 | MFS transporter | 98.4 |
| *A. baumannii* | HAV5521720.1 | MFS transporter | 94.42 |
| *A. pittii* | WP_017400389.1 | MFS transporter | 93.3 |
| *A. oleivorans* | WP_199988215.1 | MFS transporter | 92.63 |
| *A. nosocomialis* | WP_233930739.1 | MFS transporter | 92.63 |
| *A.rongchengensis* | WP_120384603.1 | MFS transporter | 90.18 |
| *A. bereziniae* | WP_151779285.1 | MFS transporter | 85.43 |
| *A. guillouiae* | WP_004817260.1 | MFS transporter | 85.43 |
| *A. baylyi* | WP_004921875.1 | MFS transporter | 85.2 |
| *A. haemolyticus* | WP_134251606.1 | MFS transporter | 84.86 |
| *A. soli* | WP_004935863.1 | MFS transporter | 84.72 |
| *A. ursingii* | WP_125625105.1 | MFS transporter | 84.53 |
| *A. soli* | WP_111811999.1 | MFS transporter | 84.49 |
| *A. guerrae* | WP_120368681.1 | MFS transporter | 84.38 |
| *A. ursingii* | WP_151831866.1 | MFS transporter | 83.86 |
| ^▲^referred to the amino acid sequence of BIT33_RS14560. | | | |

| **Supplementary Table S2 \|** DNA sequences of the chosen strains used for the construction of phylogenetic trees. | | |
| --- | --- | --- |
| Strain | Accession number | Identity(%) |
| *A. baumannii* ATCC19606 | CP058289.1^▲^ | 100 |
| *A. baumannii* ATCC17961 | CP065432.1 | 100 |
| *A. baumannii* AC1633 | CP059300.1 | 98.07 |
| *A. baumannii* A118 | CP059039.1 | 98 |
| *A. baumannii* AR_0088 | CP027530.1 | 97.55 |
| *A. baumannii* 40288 | CP077801.1 | 97.55 |
| *A. baumannii* 10324 | CP023022.1 | 97.4 |
| *A. baumannii* AB046 | CP037872.1 | 96.88 |
| *A. baumannii* VB16141 | CP040050.1 | 96.81 |
| *A. baumannii* Ab-C63 | CP051866.1 | 96.66 |
| *A. baumannii* AR_0052 | CP027183.1 | 96.51 |
| *A. baumannii* AB031 | CP009256.1 | 96.36 |
| *A. baumannii* AB048 | CP037870.1 | 96.14 |
| *A. baumannii* ATCC17978 | CP059041.1 | 95.84 |
| ^▲^referred to the DNA sequence of BIT33_RS14560. | | |

**Supplementary** **Table S3 |** Sources and proportion of 62 *A. baumanii* isolates used in this study.

| **Source** | **Number of strains (N)** | **Proportion (%)** |
| --- | --- | --- |
| sputum | 25 | 40.3 |
| blood | 4 | 6.5 |
| catheter | 3 | 4.8 |
| abdominal dropsy | 3 | 4.8 |
| urine | 2 | 3.2 |
| bile | 2 | 3.2 |
| secretion | 1 | 1.6 |
| BALF | 1 | 1.6 |
| drain | 1 | 1.6 |
| pleural effusion | 1 | 1.6 |
| NA | 19 | 30.6 |
| total | 62 | 100 |

BALF, bronchoalveolar lavage fluid; NA, not acquired.

**Supplementary Table S4 |** Analytical results of Spearman's rank correlation coefficient for biofilm biomass rank and △CT value of *BIT33_RS14560*.

|  |  |  | **△CT** | **Biofilm rank** |
| --- | --- | --- | --- | --- |
| Spearman's rho | △CT | Correlation Coeffecient | 1.000 | -0.569 |
|  |  | Sig. (2-tailed) | - | 0.000 |
|  |  | N | 62 | 62 |
|  | Biofilm rank | Correlation Coeffecient | -0.569 | 1.000 |
|  |  | Sig. (2-tailed) | 0.000 | - |
|  |  | N | 62 | 62 |

Correlation is significant at the 0.01 level (2-tailed). △CT is defined as the CT value of the target gene minus that of the reference *rpoB* gene.
